# Correlated levels of cerebrospinal fluid pathogenic proteins in drug-naïve Parkinson’s disease

**DOI:** 10.1186/s12883-019-1346-y

**Published:** 2019-06-04

**Authors:** Hidetomo Murakami, Takahiko Tokuda, Omar M. A. El-Agnaf, Takuma Ohmichi, Ayako Miki, Hideaki Ohashi, Yoshiyuki Owan, Yu Saito, Satoshi Yano, Tamao Tsukie, Takeshi Ikeuchi, Kenjiro Ono

**Affiliations:** 1 Department of Neurology, School of Medicine, Showa University, 1-5-8 Hatanodai, Shinagawa-ku, Tokyo, 142-8666 Japan; 2 Department of Molecular Pathobiology of Brain Diseases, Kyoto Prefectural University of Medicine, 465 Kajii-cho, Kawaramachi-Hirokoji, Kamikyo-ku, Kyoto, 602-8566 Japan; 3 Neurological Disorders Research Center, Qatar Biomedical Research Institute (QBRI), Hamad Bin Khalifa University (HBKU), Education City, Qatar Foundation, P.O. Box 5825, Doha, Qatar; 4 Department of Neurology, Kyoto Prefectural University of Medicine, 465 Kajii-cho, Kawaramachi-Hirokoji, Kamikyo-ku, Kyoto, 602-8566 Japan; 5 Department of Molecular Genetics, Brain Research Institute, Niigata University, 1-757 Asahimachi, Chuo-ku, Niigata, 951-8585 Japan

**Keywords:** Parkinson’s disease, α-Synuclein, Oligomer, Amyloid β-protein (1–42), Tau protein, Clinical symptom

## Abstract

**Background and aim:**

Toxic oligomeric α-synuclein (αS; O-αS) has been suggested to play a central role in the pathogenesis of Lewy body diseases such as Parkinson’s disease (PD). Cerebrospinal fluid (CSF) levels of αS, O-αS, total and phosphorylated tau, and amyloid β 1–42 (Aβ1–42) are thought to reflect the pathophysiology or clinical symptoms in PD. In this study, we examined correlations of the CSF levels of these proteins with the clinical symptoms, and with each other in drug-naïve patients with PD.

**Methods:**

Twenty-seven drug-naïve patients with PD were included. Motor and cognitive functions were assessed using the Unified Parkinson’s Disease Rating Scale (UPDRS), Montreal Cognitive Assessment (MoCA), and Neurobehavioral Cognitive Status Examination (COGNISTAT). CSF levels of total αS, O-αS, Aβ1–42, total tau and tau phosphorylated at threonine 181 (P-tau181p) were measured. CSF levels of these proteins were compared with clinical assessments from the UPDRS, MoCA and COGNISTAT using Spearman correlation analysis. Spearman correlation coefficients among CSF protein levels were also evaluated.

**Results:**

CSF levels of αS were negatively correlated with UPDRS part III (motor score) (*p* < 0.05) and bradykinesia (*p* < 0.01), and positively correlated with COGNISTAT subtest of judgement (*p* < 0.01) and CSF levels of Aβ1–42 (*p* < 0.001), total tau (*p* < 0.001) and P-tau181p (*p* < 0.01). Lower CSF levels of Aβ1–42, total tau and P-tau181p were significantly related to worsening of some motor and/or cognitive functions. The CSF level of O-αS showed no correlation with any motor and cognitive assessments or with CSF levels of the other proteins.

**Conclusion:**

CSF levels of αS are correlated with some clinical symptoms and CSF levels of other pathogenic proteins in drug-naïve PD patients. These correlations suggest a central role for interaction and aggregation of αS with Aβ1–42, tau, and phosphorylated tau in the pathogenesis of PD. Although O-αS has been shown to have neurotoxic effects, CSF levels do not reflect clinical symptoms or levels of other proteins in cross-sectional assessment.

**Electronic supplementary material:**

The online version of this article (10.1186/s12883-019-1346-y) contains supplementary material, which is available to authorized users.

## Background

Parkinson’s disease (PD) is a common neurodegenerative disorder, but diagnosis based on assessment of clinical symptoms and radiological findings can be difficult, especially in the early phase. Therefore, biomarkers reflecting the pathophysiology of PD are required, and pathogenic proteins related to neurodegeneration in cerebrospinal fluid (CSF) may be candidates as such biomarkers. The pathological hallmark of PD is the presence of Lewy body, that is, abnormal aggregates of α-synuclein (αS) in the brain. Recently, oligomeric α-synuclein (O-αS) has been shown to have neurotoxic effects, and such oligomers may play a central role in the pathogenesis of PD [1–3]. Most studies of CSF levels of total αS and O-αS have shown that total αS is decreased in PD patients compared to normal controls [4, 5], while O-αS is increased in PD [4–6]. Therefore, CSF levels of total αS and O-αS may reflect progression of the pathological background and clinical symptoms in patients with PD and may be candidate biomarkers for PD.

In Alzheimer’s disease (AD), the CSF level of amyloid β (Aβ)1–42 (Aβ1–42) is decreased and that of tau is increased [7]. These proteins are established biomarkers for diagnosis of AD, and PD can present with AD pathology such as senile plaques and neurofibrillary tangles [8]. Previous studies comparing CSF levels of these potential biomarkers between PD cohorts and controls have shown that Aβ1–42, total tau and phosphorylated tau (phosphorylated at threonine 181; P-tau181p) are significantly decreased in PD [9]. These findings suggest that the levels of these proteins in CSF are related to the pathological background of PD patients. Tau plays an important role in the structural integrity of the neuron, and phosphorylation of tau reduces its binding affinity for microtubules and causes self-aggregation, which results in neuronal damage [10]. In contrast, CSF levels of these proteins in PD are correlated with each other. For example, total αS levels are positively correlated with the levels of total tau [4, 5], phosphorylated tau [4, 5] and Aβ1–42 [4]. These correlations suggest interactions between these proteins.

Many studies in PD patients at various clinical stages and under different medication have shown correlations between CSF levels of the candidate biomarker proteins and clinical symptoms, but the results have not necessarily agreed among studies. Correlations of CSF levels of these proteins with motor and cognitive functions are rarely compared in drug-naïve PD patients. Therefore, in this study, we examined correlations of motor and cognitive functions, and CSF protein levels only in drug-naïve PD patients.

## Material and methods

### Patients

Twenty-seven patients with de novo PD were enrolled in the study. Diagnosis of PD was made according to MDS clinical diagnostic criteria for PD [11]. Patients were first diagnosed based upon their clinical history and neurological findings before medication. The diagnosis of PD was confirmed when motor symptoms improved with dopaminergic medication. Clinical and CSF data for this study were obtained within the two months before medication. MRI findings in all participants indicated no abnormal intensity area or focal atrophy suggesting parkinsonism other than PD. Patients with dementia with Lewy bodies (DLB) based on the fourth consensus report of the DLB consortium [12] were excluded. None of the participants had apparent cognitive impairment before and at least 1 year after onset of parkinsonism. Therefore, our participants had a spectrum from PD with normal cognition to PD with dementia (PDD) through PD with mild cognitive impairment (PD-MCI). To exclude the effects of medication on clinical assessments and CSF protein levels, we focused on drug-naïve PD patients. None of the patients had any other disease or were taking medications that might affect motor and cognitive function.

### Collection of CSF

Lumbar puncture was performed at the vertebral interspaces of L3-L4 or L4-L5 in a fasting state before treatment. The first 5 ml of CSF was used for routine examination, and the next 10 ml was used for this study. The CSF sample was collected in a sterile polypropylene tube and centrifuged for 10 min at 400 g within 10 min after completion of lumbar puncture. The supernatant was packed in a polypropylene microtube and stored at − 80 °C until assays were performed.

### Immunoassay of total αS and O-αS in CSF

CSF total αS was analyzed using a commercially available enzyme-linked immunosorbent assay (ELISA) kit (Covance, Dedham, MA) [13, 14]. Briefly, 200 μL/well of diluted αS standards (range, 6.1–1500 pg/mL) using reconstituted stock and diluted CSF samples (200 μL/well) were added to the capture antibody-coated plate. After overnight incubation of the plate at 2–8 °C with shaking, 50 μL/well of biotinylated detector antibody was added followed by incubation for 2 h at room temperature. Diluted streptavidin horseradish peroxidase was added, and the plate was incubated at room temperature for 1 h. After washing, a mixture of 2 different chemiluminescent substrates was added and end-point luminescence was read with a microplate luminometer (SpectraMax L, Molecular Device, Tokyo). The concentration of αS was measured using standard curves with 4-parameter curve fitting.

The levels O-αS in CSF samples were measured using a single antibody sandwich ELISA, as described previously with some modification [6, 15]. An ELISA 96-well plate (Nunc Maxisorb; NUNC, Denmark) was coated by overnight incubation at 4 °C with 1 μg/mL of the anti-human αS monoclonal antibody 211 (Syn211; Thermo Fisher Scientific, IL), which recognizes amino acid residues 121–125 of human αS, in 200 mM NaHCO_3_, pH 9.6 (100 μL/well). The plate was washed with phosphate buffered saline containing 0.05% Tween20 (PBS-T) and incubated with 200 μL/well of blocking buffer, which is PBS-T containing 2.5% gelatin, for 2 h at 37 °C. After washing 200 μL/well of PBS-T, 100 μL CSF containing 1% cocktail of protease inhibitors (Protease Inhibitor Cocktail Set I; Calbiochem, Millipore, CA) and 5% heterophilic antibody inhibitor (ELISA diluent; MABTECH, Sweden) were added to each well, and then incubated at 37 °C for an additional 3 h. Biotinylated 211 monoclonal antibody diluted to 1 μg/mL in blocking buffer was added and incubated at 37 °C for 2 h. The plate was washed and then incubated for 1 h at 37 °C with 100 μL/well of ExtrAvidin-Peroxidase (Sigma-Aldrich, Dorset, UK) diluted 1:1000 in blocking buffer. The plate was washed and incubated with 200 μL/well of an enhanced chemiluminescent substrate (Super-Signal ELISA Femto, Pierce Biotechnology), after which chemiluminescence in relative light units was immediately measured with a microplate luminometer (SpectraMax L, Molecular Device, Tokyo). All the samples were measured in 1 plate to avoid plate-to-plate variations.

For both immunoassays, the samples were screened in blind fashion and were randomly tested.

### Immunoassay of total tau, P-tau181p and Aβ1–42

We used the multiplex xMAP Luminex platform with Innogenetics immunoassay kit-based reagents for the CSF biomarker measurements of P-tau181p, total tau, and Aβ1–42 (INNO-BIA ALzBio3, Ghent, Belgium) [16, 17]. Briefly, the Innogenetics kit reagents includes well-characterized capture monoclonal antibodies specific for Aβ1–42 (4D7A3), total tau (AT120), and P-tau181p (AT270), each chemically bonded to unique sets of color-coded beads, and analyte-specific detector antibodies (HT7, 3D6). All analyses were performed in duplicate in accordance with the platform for Japanese ADNI biomarker measurement [18] and while blinded to clinical data, and batch-wise on one occasion, by a board-certified laboratory technician at the Department of Molecular Genetics, Niigata University. Intra-assay coefficients of variation were below 10%.

### Assessment of clinical symptoms

The Unified Parkinson’s Disease Rating Scale (UPDRS) [19] was used for assessment of motor function. This scale was assessed using the total score for Part III (motor score) and subscores for tremor (items 16, 20, 21), rigidity (item 22), bradykinesia (items 23 to 26 and 31), gait (items 13 to 15 and 29), and postural instability (items 27, 28 and 30). Cognitive function was assessed with the Montreal Cognitive Assessment (MoCA) and the Neurobehavioral Cognitive Status Examination (COGNISTAT). These neuropsychological tests were performed by examiners who were blinded to motor assessment and CSF findings. The MoCA is used to detect MCI and has a best score of 30. The COGNISTAT has 10 separate cognitive subtests: orientation, attention, language-comprehension, language-repetition, language-naming, construction, memory, calculation, similarity, and judgment [20]. In the Japanese version of the COGNISTAT, the raw score of each subtest is converted to a standard score, in which average scores in normal controls are set to 10 and standard deviations (SD) in healthy controls are set to 1 [21]. For cognitive assessment using the COGNISTAT, we used the standardized score of each subtest. We have shown that the MoCA and COGNISTAT are sensitive for detection of subtle cognitive impairment in PD patients [22].

## Statistical analysis

CSF levels of proteins were compared with clinical assessments from the UPDRS, MoCA and COGNISTAT. Correlations among CSF protein levels were also evaluated. Correlation coefficients were calculated using Spearman correlation analysis. The level of significance was *p* < 0.05 (two-tailed probability).

## Results

The participants included 14 males and 13 females. Three patients did not undergo the UPDRS and MoCA, and 8 did not take the COGNISTAT. CSF samples of all participants were watery clear and not bloody. Red blood cells were not observed microscopically in sediments of the CSF. CSF levels of P-tau181p in 2 patients were below the detection sensitivity and were excluded in analyses using P-tau181p. The number of participants in calculation of each correlation coefficient is shown in Tables 1, 2 and 3. The background of patients is shown in Table 4.

**Table 1:** Spearman correlation coefficients of each CSF protein level with patient background and motor symptoms

	Patient background				Motor symptoms					
		Age	Education	Duration from symptom onset	UPDRS part III	Tremor	Rigidity	Bradykinesia	Gait	Postural instability
Total αS	*r*	0.362	0.216	−0.314	− 0.446^*^	0.192	− 0.331	− 0.528^**^	− 0.245	−0.356
	*p*	0.0635	0.2794	0.1104	0.0289	0.3692	0.1136	0.0080	0.2482	0.0873
	n	27	27	27	24	24	24	24	24	24
O-αS	*r*	0.010	−0.036	0.203	−0.205	0.265	− 0.119	−0.224	− 0.335	−0.310
	*p*	0.9614	0.8594	0.3105	0.3356	0.2101	0.5792	0.2928	0.1095	0.1407
	n	27	27	27	24	24	24	24	24	24
Aβ1–42	*r*	0.292	0.131	−0.231	−0.085	0.140	−0.024	−0.107	− 0.160	−0.047
	*p*	0.1389	0.5150	0.2473	0.6913	0.5141	0.9104	0.6203	0.4546	0.8283
	n	27	27	27	24	24	24	24	24	24
Total tau	*r*	0.334	0.180	−0.212	−0.297	0.002	−0.195	−0.426^*^	− 0.167	−0.163
	*p*	0.0886	0.3702	0.2877	0.1581	0.9934	0.3610	0.0379	0.4346	0.4457
	n	27	27	27	24	24	24	24	24	24
P-tau181p	*r*	0.491^*^	0.081	−0.032	−0.448^*^	0.029	−0.425^*^	−0.462^*^	− 0.048	−0.338
	*p*	0.0126	0.7004	0.8804	0.0365	0.8978	0.0486	0.0303	0.8317	0.1241
	n	25	25	25	22	22	22	22	22	22

*: *p* < 0.05, **: *p* < 0.01

Abbreviations: Total αS, Total α-synuclein; O-αS, Oligomeric α-synuclein; P-tau181p, tau phosphorylated at threonine 181; UPDRS Unified Parkinson’s Disease Rating Scale

**Table 2:** Spearman correlation coefficients of each CSF protein level with cognitive assessment scores

Battery		MoCA	COGNISTAT									
Subtests		Total score	Orientation	Attention	Language-comprehension	Language-repetition	Language-naming	Construction	Memory	Calculation	Similarity	Judgement
Total αS	*r*	0.204	0.176	−0.119	0.174	0.168	0.447	0.345	0.158	0.245	0.088	0.626^**^
	*p*	0.3385	0.4706	0.6275	0.4770	0.4914	0.0551	0.1475	0.5171	0.3117	0.7213	0.0041
	n	24	19	19	19	19	19	19	19	19	19	19
O-αS	*r*	0.227	0.190	−0.161	0.171	0.146	−0.196	0.137	0.095	0.087	0.139	0.244
	*p*	0.2871	0.4354	0.5091	0.4833	0.5500	0.4216	0.5747	0.6992	0.7220	0.5711	0.3135
	n	24	19	19	19	19	19	19	19	19	19	19
Aβ1–42	*r*	0.426^*^	0.145	0.085	0.176	0.057	0.079	0.371	0.290	0.103	0.048	0.432
	*p*	0.0379	0.5530	0.7298	0.4706	0.8159	0.7493	0.1175	0.2285	0.6739	0.8441	0.0651
	n	24	19	19	19	19	19	19	19	19	19	19
Total tau	*r*	0.053	0.088	−0.227	0.111	0.268	0.461^*^	0.302	−0.030	0.288	0.116	0.547^*^
	*p*	0.8056	0.7199	0.3502	0.6514	0.2671	0.0469	0.2094	0.9016	0.2314	0.6365	0.0155
	n	24	19	19	19	19	19	19	19	19	19	19
P-tau181p	*r*	−0.292	0.051	−0.234	−0.287	−0.065	0.112	−0.054	− 0.294	−0.327	− 0.403	0.280
	*p*	0.1877	0.8459	0.3660	0.2635	0.8047	0.6678	0.8365	0.2516	0.2005	0.1085	0.2759
	n	22	17	17	17	17	17	17	17	17	17	17

*: *p* < 0.05, **: *p* < 0.01

Abbreviations: Total αS, Total α-synuclein; O-αS, Oligomeric α-synuclein; P-tau181p, tau phosphorylated at threonine 181; MoCA, the Montreal Cognitive Assessment; COGNISTAT, the Neurobehavioral Cognitive Status Examination

**Table 3:** Spearman correlation coefficients among CSF protein levels

		Total αS	O-αS	Aβ1–42	Total tau	P-tau181p
Total αS	*r*	1.000				
	*p*	–				
	n	27				
O-αS	*r*	0.074	1.000			
	*p*	0.7142	–			
	n	27	27			
Aβ1–42	*r*	0.613^***^	0.375	1.000		
	*p*	0.0007	0.0536	–		
	n	27	27	27		
Total tau	*r*	0.839^***^	−0.108	0.365	1.000	
	*p*	0.0000	0.5916	0.0609	–	
	n	27	27	27	27	
P-tau181p	*r*	0.614^**^	−0.022	0.138	0.665^***^	1.000
	*p*	0.0011	0.9170	0.5110	0.0003	–
	n	25	25	25	25	25

**: *p* < 0.01, ***:*p* < 0.001

Abbreviations: Total αS, Total α-synuclein; O-αS, Oligomeric α-synuclein; P-tau181p, tau phosphorylated at threonine 181

**Table 4:** Patient background

Male: Female	14:13	
Age (years)	72.3 ± 9.2	(range 47–84)
Education (years)	12.2 ± 3.2	(range 3–16)
Duration from symptom onset (years)	1.3 ± 1.2	
	(< 1 year	7 persons)
	(≥ 1, < 2 years	12 persons)
	(≥ 2, < 3 years	4 persons)
	(≥ 3, < 4 years	3 persons)
	(≥ 4, < 5 years	0 person)
	(≥ 5, < 6 years	1 person)
Clinical assessment scores		
UPDRS		
Part III (motor score)	16.7 ± 8.2	(range 5–31)
Tremor	2.6 ± 2.6	(range 0–10)
Rigidity	3.0 ± 2.3	(range 0–10)
Bradykinesia	6.4 ± 4.4	(range 0–16)
Gait	2.5 ± 2.0	(range 0–7)
Postural instability	2.4 ± 1.9	(range 0–6)
MoCA	21.7 ± 5.5	(range 9–28)
COGNISTAT		
Orientation	8.9 ± 1.3	(range 6–10)
Attention	6.3 ± 3.8	(range 1–10)
Language-comprehension	8.7 ± 2.9	(range 1–10)
Language-repetition	8.6 ± 2.3	(range 4–11)
Language-naming	9.0 ± 1.6	(range 5–10)
Construction	7.5 ± 1.8	(range 4–11)
Memory	7.5 ± 1.7	(range 5–10)
Calculation	8.6 ± 2.5	(range 2–10)
Similarity	9.1 ± 1.2	(range 6–11)
Judgement	9.8 ± 1.3	(range 7–12)
CSF protein levels		
Total αS (pg/ml)	1517.3 ± 654.3	(range 573–3387)
O-αS (RLU/s)	107,019.5 ± 20,203.1	(range 74,867–161,685)
Aβ1–42 (pg/ml)	427.1 ± 159.7	(range 206.4–724.5)
Total tau (pg/ml)	38.4 ± 21.2	(range 11.2–93.9)
P-tau181p (pg/ml)	15.3 ± 6.1	(range 5.8–29.9)

Scores are shown as Mean ± Standard Deviation. Abbreviations: UPDRS, Unified Parkinson’s Disease Rating Scale; MoCA, the Montreal Cognitive Assessment; COGNISTAT, the Neurobehavioral Cognitive Status Examination; Total αS, Total α-synuclein; O-αS, Oligomeric α-synuclein; P-Tau181p, tau phosphorylated at threonine 181

**Fig. 1:**
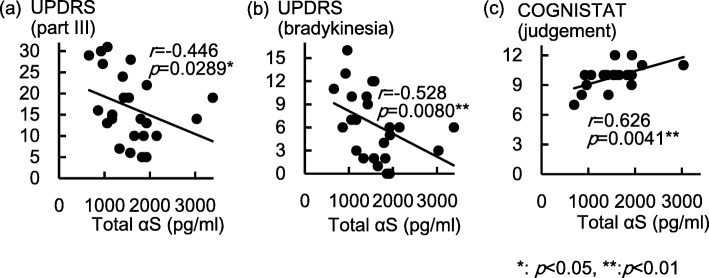
Distribution of CSF levels of total α-synuclein and clinical assessment scores. The graphs show CSF levels of total αS with **a** UPDRS part III scores, **b** UPDRS subscores for bradykinesia, and **c** COGNISTAT judgement scores. Abbreviations: Total αS, Total α-synuclein; UPDRS, Unified Parkinson’s Disease Rating Scale; COGNISTAT, Neurobehavioral Cognitive Status Examination

Spearman correlation coefficients of each CSF protein level with patient background and motor symptoms are shown in Table 1, and those with cognitive assessment scores are given in Table 2. For patient background, CSF levels of total αS, O-αS, Aβ1–42 and total tau were not correlated with age, education, or duration from symptom onset, but P-tau181p was positively correlated with age (*p* < 0.05). For clinical symptoms, CSF levels of total αS were negatively correlated with UPDRS scores for part III (*p* < 0.05, Fig. 1a) and bradykinesia (*p* < 0.01, Fig. 1b), and positively correlated with the COGNISTAT subscore for judgment (*p* < 0.01, Fig. 1c); O-αS showed no correlation with any motor and cognitive assessments; Aβ1–42 was positively correlated with MoCA score (*p* < 0.05); total tau was negatively correlated with the UPDRS subscore for bradykinesia (*p* < 0.05) and positively correlated with COGNISTAT subscores for language-naming and judgment (both *p* < 0.05); and P-tau181p was negatively correlated with UPDRS scores for part III, rigidity, and bradykinesia (all *p* < 0.05).

**Fig. 2:**
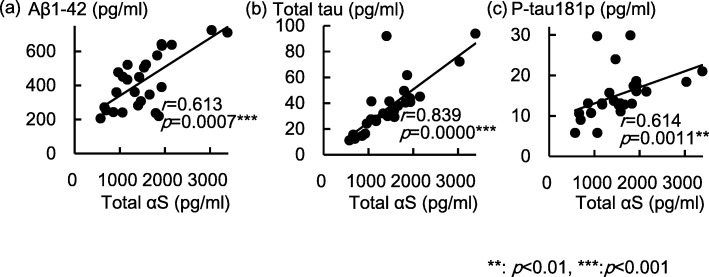
Distribution of CSF levels of total α-synuclein and CSF protein levels. The graphs show CSF levels of total αS with CSF levels of **a** Aβ1–42, **b** total tau, and **c** P-tau181p. Abbreviations: Total αS, Total α-synuclein; Aβ1–42, amyloid β (Aβ)1–42; P-tau181p, tau phosphorylated at threonine 181; O-αS, oligomeric α-synuclein

Spearman correlation coefficients among CSF protein levels are shown in Table 3. CSF levels of total αS were positively correlated with those of Aβ1–42 (*p* < 0.001, Fig. 2a), total tau (*p* < 0.001, Fig. 2b), and P-tau181p (*p* < 0.01, Fig. 2c). CSF levels of O-αS showed no correlation with those of any other proteins.

## Discussion

The results of this study showed that CSF levels of total αS are negatively correlated with UPDRS part III and bradykinesia scores, which indicates that these motor functions worsen as the CSF level of total αS decreases. This result agrees with those from the Parkinson’s Progression Marker’s Initiative (PPMI) study, which showed that the CSF αS level is negatively correlated with MDS-UPDRS part III (motor score) and the Hoehn and Yahr scale in drug-naive PD patients [23]. Therefore, total αS in CSF decreases with progression of PD, which may be due to aggregation and intracellular accumulation of αS [4, 5, 24]. In the present study, the CSF level of total αS showed a positive correlation with the COGNISTAT subtest of judgment, which suggests worsening judgment as the CSF total αS level decreases. This subtest requires patients to explain how to overcome a presented abnormal and critical situation; that is, it evaluates frontal/executive function. Our results are consistent with those in Skogseth et al., in which it was also found that a lower CSF αS level was associated with reduced performance on executive function [25].

Although O-αS is toxic and mediates neural damage [1–3], no clinical symptom correlated with the CSF level of O-αS in this study. In most studies of CSF O-αS, the level has been shown to be higher in PD patients than in normal controls [4–6]. Our results suggest that CSF levels of O-αS do not necessarily correlate with clinical symptoms or the extent of neuronal damage in a cross-sectional study. There may be a time lag between dynamics of CSF levels of O-αS and changes in clinical symptoms. On the other hand, O-αS accumulates in the brain, and therefore, intracellular O-αS may affect clinical symptoms in PD patients and may be related to motor and cognitive functions. However, the intraneuronal level of O-αS cannot be measured in a clinical setting.

The CSF levels of total tau and P-tau181p as well as total αS were negatively correlated with bradykinesia scores. Bradykinesia is the earliest and most fundamental motor symptom in PD [11]. These correlations show that as the disease progresses, CSF levels of total tau, P-tau181p and total αS concurrently decrease. Similar associations of CSF total tau and αS with motor severity in de novo PD patients were found in the PPMI study [23]. In our study, CSF levels of total tau and P-tau181p also showed strong positive correlations with that of total αS. Waxman et al. showed that αS can induce aggregation of tau, and that phosphorylation of these aggregates then progresses in vivo [26]. Guo et al. showed similar results for αS cross-seeding tau aggregation in vivo [27]. Ishizawa et al. found tau- and phosphorylated tau-positive Lewy bodies in immunostaining of postmortem brain in Lewy body disease patients [28]. These findings suggest that tau and phosphorylated tau both aggregate with αS, which explains the correlation of their decreased CSF levels. Correlations of CSF levels of both total tau and P-tau181p with total αS were stronger than those with bradykinesia. This suggests that the interaction between these proteins is primary, and that bradykinesia then emerges. In AD, the CSF level of tau is increased, which may be due to releases of tau protein from damaged neurons into the CSF [7]. Although PD and AD are both neurodegenerative disease, the dynamics of tau may differ in the two diseases.

In the present study, the CSF level of Aβ1–42, an AD biomarker, was positively correlated with MoCA score, which suggests that cognitive function deteriorates as the CSF Aβ1–42 level decreases. In AD, a decrease in CSF Aβ1–42 is thought to be due to its aggregation in the brain [29]. Therefore, our results suggest that cognitive function deteriorates as aggregation of Aβ1–42 progresses. However, the CSF level of Aβ1–42 showed a stronger positive correlation with that of total αS in the present study. This suggests that αS and Aβ1–42 interact. It was previously shown that Aβ enhances αS accumulation and neuronal deficits in vivo [30], and an in vitro study indicated that Aβ and αS might interact directly to form pore-like oligomers that contribute to neurodegeneration [31]. We previously showed that αS and Aβ1–42 act as seeds and affect each other’s aggregation pathways in vivo, suggesting a molecular interaction between AD and PD [32]. Co-existence of αS and Aβ1–42 deposits in human brain is found in pathological studies, and PD patients present with an AD pathology of amyloid deposition, such as senile plaque [8]. Lewy bodies are found in 56.8% of AD cases [33], Aβ deposits are distributed throughout the medial temporal lobe in DLB patients [34], and Aβ deposition is associated with enhanced cortical αS lesions in postmortem brain of PD patients [35]. Therefore, αS and Aβ1–42 co-aggregate and their CSF levels concurrently decrease. The stronger correlation of the CSF level of Aβ1–42 with the CSF level of total αS compared to that with the MoCA total score suggests that the interaction between these proteins is the primary event, with subsequent emergence of cognitive deterioration.

In the PPMI study, a correlation between CSF levels of αS and Aβ1–42 was not observed [23]. The PPMI study included earlier drug-naïve PD patients diagnosed within no more than 2.6 years (median 0.4 years) from onset. In contrast, the present study included many PD patients with a longer duration from onset. Therefore, we calculated the correlation coefficients in patients with a shorter disease duration. The significant correlation between total αS and Aβ1–42 was preserved in patients with a disease durations of < 1 year (*n* = 7, r = 0.929, *p* < 0.01), but not in patients with a disease durations of ≥1 year (*n* = 20, r = 0.284, *p* = 0.225). On the other hand, CSF level of total αS correlated with total tau (n = 20, r = 0.767, *p* = 0.000) and P-tau181p (*n* = 18, r = 0.564, *p* = 0.015) in patients with a disease durations of ≥1 year and these results agree the PPMI study [23]. Difference in correlation of CSF level of total αS between Aβ1–42 and total and phosphorylated tau proteins in patients with a disease duration of ≥1 year suggests dynamics of these proteins differ. A possible hypothesis to explain this difference is as follows. In earlier phase which was shown to be a disease duration of < 1 year in our study, these intracellular proteins aggregate as mentioned above. In later phase, neurocytotoxic change progress to promote the aggregation of extracellular Aβ1–42, and the aggregates of Aβ1–42 pull out αS from neurons. Consequently, correlation of CSF levels of αS and Aβ1–42 becomes unclear. However the turning point of the earlier to later phase may differ in each patients and/or cohorts due to unknown factors. Majority of participants in the PPMI study may have the pathophysiology of the later phase as hypothesized above. The reasons for the difference in correlations between our study and the PPMI study are uncertain. Future studies in other cohorts are required to investigate this correlation and elucidate the pathology of PD.

This study includes only drug-naïve PD patients and the effect of medication is eliminated. Our results support a central role for interaction and aggregation of αS with Aβ1–42, tau, and phosphorylated tau in the pathogenesis of PD. However, the study has limitations. The number of patients is relatively small and we did not include a normal control group. A larger number of participants and control group would have made our conclusions more comprehensive and meaningful. However, recruitment of patients who agree to lumbar puncture is difficult, since most patients with parkinsonism want to be examined without lumbar puncture, and we could not recruit healthy volunteers who agreed to undergo lumbar puncture. Consequently, only 27 patients agreed to participate in the study. However, therapies targeting pathogenic proteins are under development, and knowledge of the relationship between pathogenic proteins and clinical symptoms is important. Therefore, we believe our report is significant and contributes to understanding of the pathophysiology of PD.

## Conclusions

CSF levels of αS correlate with some clinical symptoms and with CSF levels of Aβ1–42, total tau and P-tau181p in drug-naïve PD patients. These correlations suggest a central role for interaction and aggregation of αS with Aβ1–42, tau, and phosphorylated tau in the pathogenesis of PD. Although O-αS has been shown to have neurotoxic effects, CSF levels do not reflect clinical symptoms or levels of other proteins in cross-sectional assessment.

## Additional file

Additional file 1: All data used during this study. (XLSX 13 kb)

## Data Availability

All data used during this study are included in the Additional file 1.

## Abbreviations

AD: Alzheimer’s Disease
Aβ: amyloid Amyloid β
COGNISTAT: the The Neurobehavioral Cognitive Status Examination
CSF: Cerebrospinal Fluid
DLB: Dementia with Lewy Bodies
ELISA: Enzyme-Linked Immunosorbent Assay
MCI: Mild Cognitive Impairment
MoCA: Montreal Cognitive Assessment
O-αS: Oligomeric α-synuclein
PD: Parkinson’s Disease
PDD: Parkinson’s Disease with Dementia
PD-MCI: Parkinson’s Disease with Mild Cognitive Impairment
PPMI: Parkinson’s Progression Marker’s Initiative
P-tau181p: tau Tau phosphorylated at threonine 181
SD: Standard Deviation
UPDRS: the The Unified Parkinson’s Disease Rating Scale
αS: α-synuclein
